# Exploring sagebrush leaf microbial metagenomes from deep, host-derived sequencing

**DOI:** 10.1128/spectrum.02198-25

**Published:** 2026-03-31

**Authors:** Adedotun Adedayo Arogundade, Carlos Dave C. Dumaguit, Anthony Melton, Sven Buerki, Leonora S. Bittleston

**Affiliations:** 1 Department of Biological Sciences, Boise State University 166917, Boise, Idaho, USA; 2 Department of Nutrition and Integrative Physiology, University of Utah 228463, Salt Lake City, Utah, USA; 3 Department of Biology, Chemistry, Mathematics and Computer Sciences, University of Montevallo 5578, Montevallo, Alabama, USA; Ruhr-Universitat Bochum, Bochum, Germany

**Keywords:** microbial metagenomics, metagenome-assembled genomes (MAGs), sagebrush, phyllosphere microbiome, microbial bioinformatics, leaf microbiome, plant-host derived microbial profiling, plant-associated microbial communities, functional annotation, Illumina reads, microbial ecology

## Abstract

Advanced sequencing technologies and improvements in bioinformatics have provided a new way to study plant-associated microbial communities, including the use of host genomic sequencing. Our study focuses on the leaf microbiome of basin big sagebrush (*Artemisia tridentata* subsp. *tridentata*), a foundational shrub of western North America. We analyzed Illumina shotgun sequences from sagebrush leaves to investigate the metagenomes of leaf-associated microbes that were sequenced alongside their plant hosts. We aimed to profile the leaf microbiome across different sample sources (magenta box, greenhouse, and field/wild), reconstruct metagenome-assembled genomes (MAGs) where possible, and investigate functional gene annotations of the resulting MAGs, specifically with regard to the potential metabolism of sagebrush chemicals. To achieve this, Illumina shotgun sequence reads (containing both host and associated microbial reads) were mapped to the reference genomes of *Artemisia tridentata*, *Artemisia annua*, and the human reference genome to remove plant host and human-associated sequences. Host-cleaned reads were then analyzed using microbial metagenomics techniques. Taxonomic profiling revealed that *Phyllobacterium* and *Sphingomonas* were the most abundant microbial genera in greenhouse-grown plants, with very little variation among the samples. Wild, field-collected samples were much more variable and were dominated by *Klebsiella* and *Aureobasidium* species. From the co-assembly of greenhouse samples, we reconstructed two high-quality MAGs (a *Phyllobacterium* species and a *Sphingomonas* species) with >98% completion and <1% contamination. Functional annotation of these MAGs uncovered genes associated with the degradation and metabolism of camphor and other essential oils such as pinene, geraniol, and limonene, which are part of sagebrush leaf chemistry.

**IMPORTANCE:**

Big sagebrush (*Artemisia tridentata*), the foundation species of the sagebrush steppe, has broad ecological importance because its evergreen leaves offer nutrients and shade that facilitate the establishment of diverse understory plants in arid environments. Sagebrush leaves contain various secondary metabolites, including terpenoids, flavonoids, and phenolic compounds. These chemicals contribute to the plant’s defense mechanisms against herbivores and pathogens. Despite this, sagebrush hosts diverse bacterial and fungal communities. We found that the microbial metagenome-assembled genomes (MAGs) we recovered contained genes that have the potential to degrade some of the chemical compounds in sagebrush leaves that could inhibit the growth of other microbes. This is the first study to mine plant genome data using host-derived sequences to generate microbial MAGs. Our results showed that MAGs can be recovered from plant host-derived sequence data, providing a new way to explore the identity and functional capabilities of difficult-to-culture microbes.

## INTRODUCTION

The traditional method for characterizing microbes and sequencing their genomes begins with the isolation of individual strains from laboratory cultures (1, 2). However, many microbes are difficult to culture because their natural habitat is too complex to be reproduced in the laboratory or because they rely on other species to successfully grow. This has limited the scope of microbial studies and has left many microbes uncharacterized (3, 4). However, metagenomic sequencing provides a culture-independent approach to studying complex communities of microbes (5, 6). This culture-independent approach supports taxonomic and functional profiling of microbes, as well as reconstruction of metagenome-assembled genomes (MAGs). A MAG refers to a collection of similar scaffolds that are grouped together from a metagenome assembly based on tetranucleotide frequencies, abundances, complementary marker genes (7), taxonomic alignments (8), and codon usage (9) to represent a microbial genome.

The plant microbiome, defined as the community of microorganisms living in association with a plant, is increasingly recognized as essential to plant health, ecosystem functioning, and sustainable agriculture (10). Microbial assembly in this habitat is governed by complex interactions among microbes, their plant hosts, and the environment (11, 12). Numerous recent studies of the plant microbiome have explored microbial functional potential and composition using metagenomics techniques. For example, analyzing microbial communities across different plant species and conditions has helped to uncover how microbial assemblage and function vary based on factors like geography, climate, and plant genotype (13–15). Most plant metagenomics studies wash the microbes from the leaves and concentrate them prior to DNA extraction (16). A much rarer approach is to start with whole-leaf DNA sequencing and remove the host plant DNA to focus on the microbial component (17). This approach allows us to use existing whole-genome sequencing for plant genomes to uncover additional information about their microbial associations and thus their extended phenotype (17).

Our study focuses on the leaf microbiome of basin big sagebrush (*Artemisia tridentata* subsp. *tridentata;* Asteraceae), a foundational shrub species that dominates much of western North America (18). A reference-quality genome assembly was recently completed for this species, and deep sequencing is available across multiple individuals (19, 20). The ecological attributes of sagebrush affect community composition, ecosystem processes, and wildlife habitat over vast portions of western North America (18). Its evergreen leaves offer nutrients and shade that facilitate the establishment of diverse understory plants even in arid environments (21). Sagebrush foliage is relatively unpalatable to livestock and other large herbivores due to high levels of terpenes and tannins, thereby buffering more palatable understory plants from intensive grazing and allowing sensitive species like perennial bunchgrasses to persist (18). Greater sage-grouse, pygmy rabbits, pronghorn, and mule deer rely on sagebrush for shelter and their primary winter food source (21). Unfortunately, sagebrush ecosystems are critically threatened by wildfires, invasive species, climate change, land conversion, and urban development (22), which has resulted in the loss of more than 50% of the historic sagebrush range (23). The major ecological role of sagebrush underscores the need to protect the remaining intact sagebrush communities through ecological restoration and sustainable management in order to conserve biodiversity and ecosystem services across the western USA (18).

Although there is minimal research into the sagebrush phyllosphere microbiome (in the above-ground parts of plants), recent sequencing and culture-based studies have shown that, despite the high levels of terpenoids, phenolics, and other antibiotic metabolites in the foliage, sagebrush plants host diverse bacterial and fungal communities in and on their leaves (24, 25). Therefore, it is likely that sagebrush microbes are able to degrade many of the chemical compounds in the leaves, while other species are likely inhibited by the leaf-associated chemicals. Sagebrush-associated microbes could potentially confer stress tolerance, nutrient acquisition, and pathogen protection benefits to their host plant, as has been found for other plant species (26, 27). Furthermore, establishing linkages between sagebrush chemistry and microbiome composition might offer new insights into plant-microbe coevolution. Reports have shown that terpene profiles vary across sagebrush subspecies and populations, which may drive corresponding shifts in associated microbiomes (28). Thus, comparative analyses of microbiomes among sagebrush chemotypes could uncover specialist microbial taxa adapted to particular compounds.

Culture-based experiments and amplicon sequencing (e.g., 16S for bacteria and ITS for fungi) have provided insight into the diversity and profile of microbes associated with the sagebrush phyllosphere (24, 25). However, these analyses are limited compared to shotgun-based metagenomics because they do not give insight into functional capabilities (29). In addition to taxonomic profiling, shotgun metagenomics provides a platform for genome-focused functional analysis if MAGs can be recovered (30). Characterizing genomes of prevalent or specialized sagebrush-associated microbes may reveal metabolic pathways involved in the detoxification or utilization of terpenoids, polyphenols, or other secondary metabolites that are produced by sagebrush. Furthermore, advances in sequencing technologies and bioinformatics analyses have led to the development of methods that allow more complete analyses of microbes that are not yet cultivated (31).

The objectives of this study were to profile the microbial taxa associated with basin big sagebrush leaves across different sample sources, reconstruct MAGs where possible, and explore the potential functions of the resulting MAGs. We asked the following questions: (i) Which microbial taxa can we profile from deeply sequenced sagebrush shotgun reads? (ii) How does host genotype or phenotype affect microbial composition and diversity in greenhouse and wild settings? (iii) Are there differences in the leaf-associated microbial community composition between greenhouse-grown vs wild plants? (iv) Can we reconstruct draft microbial genomes (MAGs) directly from sagebrush sequencing data? And if yes, (v) do these MAGs possess genes capable of metabolizing sagebrush chemicals?

## MATERIALS AND METHODS

### Genomic samples

We used existing sequence data from previous studies of sagebrush by researchers at Boise State University who developed *in vitro* propagation protocols to assemble the genome (32) and propagated seedlings from different populations (33). These data are divided into three categories based on the environment in which the plants were grown prior to sequencing. Sagebrush has a genome size of ~4.2 Gb (20).

#### Sterile magenta box

We analyzed Illumina HiSeq paired-end sequencing data from two plants grown in axenic or sterile conditions (32) that were sequenced at ~160× read depth (20). We expect that these plants should have little to no plant-associated microbes.

#### Greenhouse

Twelve plants were grown in a controlled, greenhouse environment. DNA was extracted from plant leaf tissues and was sent for Illumina HiSeq paired-end sequencing at ~20× read depth. This includes three groups of plants with origin locations in Idaho, Utah, and Nevada (34). Each region has four representative plants. Previous studies have shown that these plants have varying genotypes, and they are the focus of our research question to investigate the relationship between microbial composition and host genotypes.

#### Field

Illumina HiSeq data at ~10–12× read depth from 14 wild plants were also analyzed. All plants are likely to be the same general genotype, as they came from a single population but with different phenotypes, specifically, leaf-dropping phenotypes. Samples 14, 15, 26, 29, 34, 35, and 36 drop their leaves early, while samples 2, 21, 24, 3, 32, 33, and 8 drop their leaves late. These data enabled us to compare the diversity of microbes between wild plants with different phenotypes and also between wild plants and those grown in the greenhouse.

### DNA extraction and sequencing

As reported by Melton et al. (20), DNA extraction for all the samples was done using the QIAGEN DNeasy Plant Mini Kit (Hilden, Germany; catalog # 69204) using approximately 20 mg of plant leaf tissue per sample. The samples were then processed for library preparation and Illumina HiSeq (San Diego, CA, USA) whole-genome paired-end sequencing (2 × 150 bp) by Azenta Life Sciences (New Jersey, NJ, USA), formerly Genewiz Inc.

### Bioinformatic workflow

#### Preprocessing (quality assessment and removal of host reads)

**Fig 1:**
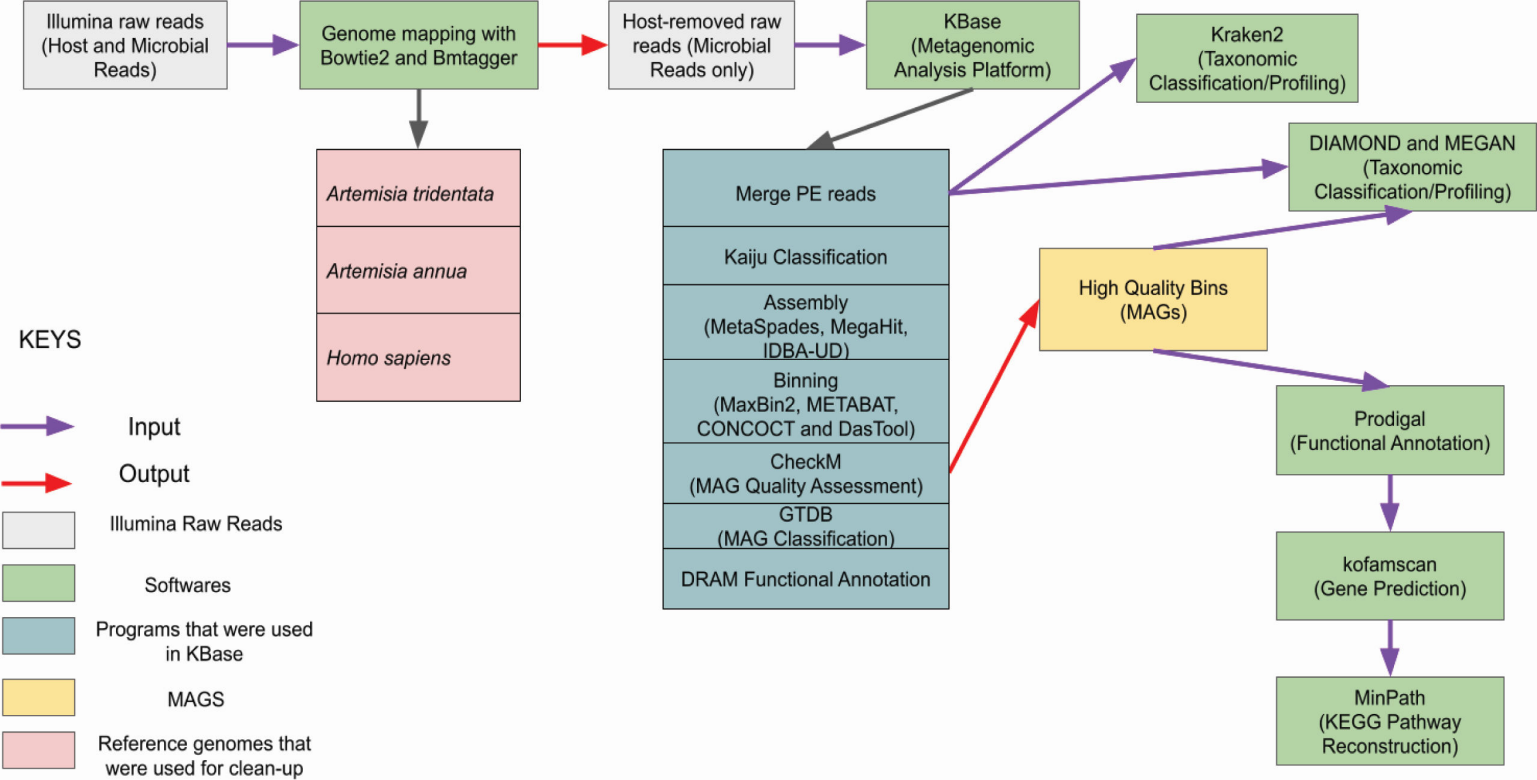
Overview of the bioinformatic workflow for the recovery of metagenome-assembled genomes from host-derived sequences of basin big sagebrush *Artemisia tridentata* subsp. *tridentata*

The overview of the bioinformatic workflow used in this study is shown in Fig. 1. Preprocessing is the first step in any metagenomic analysis: removing adapter sequences, filtering low-quality nucleotides/reads, and generating quality assessment metrics. This was done using FastQC (35), and the Illumina sequences with Phred scores <20 were filtered with Trimmomatic (36). Our sequences were host-derived as they were extracted from plant leaf tissues and contained sequences from the host and the collective DNA of resident microbial communities (17), and with our focus on the associated microbial reads, we consider host genomes as contaminants that could impact our analyses. Using Bowtie2 (37), we mapped the trimmed reads to the reference genome of *Artemisia tridentata* subsp. *tridentata* and used Samtools to filter the alignment file to retain the unmapped reads, which are considered “non-host” reads as described by Roman-Reyna et al. (17). However, downstream analysis showed that there were still sagebrush reads in our “non-host” reads; therefore, we used BMTagger (38), a tool specifically designed to remove the human genome from metagenomic data sets but that can be adapted to remove other host genomes. In order to improve the clean-up process, we indexed reference genomes from *Artemisia tridentata* subsp. *tridentata*, *Artemisia annua*, and the human reference genome GRCh38/hg38 (NCBI Accession # GCA_000001405.15) and mapped them with our data sets. Table 1 shows sample information and the amount of “host” or “human” reads that were removed from each of our sequencing data sets. See the Supplementary Information for additional bioinformatic tools that were used but not included in the final workflow.

**TABLE 1:** Sample information including amount of host or human reads that were removed from the sequencing data

Sample ID	Category	Initial gzipped file size	Approximate size of host/human data removed from cleaned reads			Final gzipped file size	Number of cleaned PE raw reads (basepairs)
			*A. tridentata*	*A. annua*	*Homo sapien*		
IDT3	Magenta	38.0 Gb	24.0 Gb	4.4 Gb	970 Mb	8.4 Gb	25,738,784
UTT2	Magenta	58.0 Gb	34.0 Gb	4.6 Gb	1.31 Gb	18.2 Gb	55,350,386
29-ID	Greenhouse	4.4 Gb	2.2 Gb	658 Mb	0 Mb	1.48 Gb	4,207,818
143-ID	Greenhouse	3.6 Gb	1.83 Gb	550 Mb	0 Mb	1.29 Gb	3,683,012
163-ID	Greenhouse	3.4 Gb	1.84 Gb	540 Mb	0 Mb	1.11 Gb	3,158,346
192-ID	Greenhouse	3.4 Gb	1.66 Gb	456 Mb	0 Mb	1.31 Gb	3,717,942
246-NV	Greenhouse	4.2 Gb	2.20 Gb	602 Mb	0 Mb	1.58 Gb	4,490,656
326-NV	Greenhouse	3.6 Gb	1.98 Gb	576 Mb	0 Mb	1.15 Gb	3,267,482
379-NV	Greenhouse	3.8 Gb	2.02 Gb	560 Mb	0 Mb	1.29 Gb	3,666,774
399-NV	Greenhouse	4.0 Gb	2.40 Gb	580 Mb	0 Mb	1.21 Gb	3,440,062
422-UT	Greenhouse	5.8 Gb	3.20 Gb	780 Mb	0 Mb	2.02 Gb	5,794,018
443-UT	Greenhouse	4.0 Gb	2.02 Gb	562 Mb	0 Mb	1.47 Gb	4,186,566
541-UT	Greenhouse	4.0 Gb	2.20 Gb	620 Mb	0 Mb	1.25 Gb	3,558,232
579-UT	Greenhouse	3.8 Gb	1.98 Gb	564 Mb	0 Mb	1.30 Gb	3,696,426
2-Wild	Field/Wild	3.2 Gb	1.8 Gb	464 Mb	4.4 Mb	911 Mb	2,601,426
3-Wild	Field/Wild	3.4 Gb	2.0 Gb	474 Mb	3.6 Mb	907 Mb	2,590,152
8-Wild	Field/Wild	4.0 Gb	2.0 Gb	524 Mb	2.6 Mb	2.3 Gb	6,519,242
14-Wild	Field/Wild	3.0 Gb	2.0 Gb	502 Mb	1.0 Mb	853 Mb	2,436,802
15-Wild	Field/Wild	4.0 Gb	2.2 Gb	588 Mb	48 Mb	1.2 Gb	3,604,450
21-Wild	Field/Wild	4.0 Gb	2.2 Gb	540 Mb	12.8 Mb	1.3 Gb	3,559,682
24-Wild	Field/Wild	3.0 Gb	1.7 Gb	470 Mb	1.9 Mb	800 Mb	2,283,504
26-Wild	Field/Wild	3.6 Gb	2.2 Gb	548 Mb	13.4 Mb	1.0 Gb	2,960,114
29-Wild	Field/Wild	3.6 Gb	2.2 Gb	448 Mb	2.2 Mb	853 Mb	2,433,302
32-Wild	Field/Wild	3.4 Gb	2.2 Gb	510 Mb	1.5 Mb	911 Mb	2,590,152
33-Wild	Field/Wild	3.8 Gb	2.2 Gb	512 Mb	3.6 Mb	1.2 Gb	3,528,676
34-Wild	Field/Wild	3.4 Gb	1.9 Gb	488 Mb	8.4 Mb	1.1 Gb	3,125,510
35-Wild	Field/Wild	4.0 Gb	1.8 Gb	234 Mb	556 Mb	1.3 Gb	3,839,712
36-Wild	Field/Wild	3.6 Gb	2.0 Gb	518 Mb	3.0 Mb	965 Mb	2,754,048

#### Taxonomic profiling of “host”-cleaned short Illumina reads

The Department of Energy Systems Biology Knowledgebase (KBase) System was used for most of our downstream metagenomic analyses (39) with default parameters for all software. We primarily used Kaiju v.1.9.0 (the taxonomic classifier on KBase) to classify host-cleaned short reads. Kaiju uses a protein-level comparison approach, which is more sensitive than nucleotide-based methods by comparing protein sequences from the reads against a reference database (40). However, we also used other tools not found in KBase in order to enable us to compare the results of these programs.

The other taxonomic tools we used were Kraken2 and DIAMOND/MEGAN (41, 42). Kaiju and DIAMOND programs used the NCBI non-redundant protein database, and Kraken2 used the “Standard Kraken2 plus RefSeq protozoa & fungi” database. Kaiju was run using protein-level sequence classification with stringent filtering thresholds. In greedy matching mode, up to three amino acid substitutions were allowed during match extension (-e 3), and only matches with a minimum alignment score of 65 were considered for taxonomic assignment (-s 65), ensuring sufficient match length and quality. An E-value cutoff of 0.01 (-E 0.01) was specified for maximum exact match searches, corresponding to a ≤1% probability of random matches (40). DIAMOND alignments were filtered using an E-value threshold of 1e-3, and only hits with a minimum bit score of 50 were retained (42). MEGAN taxonomic assignment was performed using the LCA algorithm with a 10% top-score cutoff to minimize spurious classifications. Kraken2 classification uses exact k-mer matching, and taxonomic assignments were filtered using a confidence threshold of 0.1, requiring at least 10% of k-mers within a read to support the assigned taxon, thereby reducing spurious classifications (41).

#### Co-assembly and reconstruction of MAGs

We built co-assemblies of sequence data from plants in the same category; that is, microbial sequences from all greenhouse-grown plants were co-assembled, and those from all the field-grown plants were co-assembled. KBase provides three assembly tools: metaSPAdes v.3.15.3 (43), MEGAHIT v.1.2.9 (44), and IDBA-UD v.1.1.3 (45), and we used all three of these programs to assemble the metagenomic reads into contiguous sequences. Assembling with multiple different tools is a common way to find which one produces the best results. Using Compare Assembled Contig Distributions v.1.1.2 software on KBase, we saw that metaSPAdes has the best N50 and N75, and it produced the longest contigs. Just like with the assembly process, KBase also provides three binning programs: MaxBin2 v.2.2.4 (46), MetaBAT2 v.1.7 (47), and CONCOCT v.1.1 (48), which were all used for MAG reconstruction. The individual outputs (bins) from these programs were passed into DASTools v.1.1.2 (49) to produce high-quality, non-redundant MAGs.

#### Classification of MAGs

According to literature, the differences between read-based taxonomic profiles and MAG taxonomy arise from a combination of biological complexity and methodological bias (50). Read-based approaches capture the total pool of sequenced DNA and therefore reflect community composition across both abundant and low-abundance taxa, whereas MAG recovery is constrained by assembly and binning efficiency, which preferentially reconstructs genomes from abundant, low-diversity populations (50). Prior to classification, CheckM v.1.0.18 (51) was used to assess the quality of the MAGs, and then the Genome Taxonomy Database (GTDB) toolkit, GTDB-Tk v.1.7.0 (52), was used to classify them. We chose GTDB because it is the most common classification tool for MAGs that has been reported (52). Although traditional methods of 16S rRNA classification have helped to understand the diversity of microbes in metagenomic data sets, they are often limited in characterizing metagenome-assembled genomes because small subunits such as 16S rRNAs are poorly represented in MAGs (53, 54). To overcome this issue, GTDB-Tk uses HMMER (55) to identify a set of 120 bacterial marker genes (https://data.ace.uq.edu.au/public/gtdb/data/releases/latest/) in the reference genomes on the GTDB (56) to create multiple sequence alignments, which are then used to create an inference-based phylogenomic tree (57). The classification of the queried MAG is determined by its position on the tree. In addition to GTDB classification, we exported the fasta files of these bins from KBase and classified them using Kraken2 and DIAMOND. We found that the Kraken2 and DIAMOND results were consistent with the GTDB-generated taxonomy.

#### Gene prediction/functional profiling of MAGs

Gene prediction helps to identify the genetic potential of the recovered MAGs. This step enables the identification of functional genes and pathways relevant to plant-microbe interactions and ecosystem functioning. Distilled and Refined Annotation of Metabolism (DRAM) profiles microbial (meta)genomes for metabolisms known to impact ecosystem function (58). DRAM annotation on KBase assessed the completion of a few metabolic modules and electron transport chain complexes and also predicted the presence of carbohydrate-active enzymes in the metagenomes. We also annotated the MAGs using Prodigal (59), and the annotated genomes were then passed into KofamScan (https://github.com/takaram/kofam_scan), a command-line interface of the web-based KofamKOALA (60), a gene function annotation tool based on Kyoto Encyclopedia of Genes and Genomes (KEGG) Orthology and hidden Markov models. MinPath (61) was used to reconstruct a parsimonious KEGG (62) biological pathway using protein family predictions. This provides comprehensive reports on the metabolic modules and pathways that are found in MAGs. Sagebrush contains many secondary metabolites, including terpenes, flavonoids, phenolic compounds, and essential oils, which play essential roles in the plant’s defense against herbivores, pathogens, and environmental stressors. Several studies have reported that the major chemical compounds in sagebrush are artemiseole, p-cymene, camphor, camphene, eucalyptol, limonene, geraniol, beta-pinene, and α-pinene (63, 64). They are also essential components of the plant’s volatile organic compounds that influence the plant’s interactions with its environment, including other organisms and ecosystems. Therefore, as part of our functional annotation, we screened the MAGs for genes that code for enzymes capable of metabolizing these compounds.

#### Diversity metrics

Microbial diversity metrics were generated and analyzed using R version 4.2.1 (65). Community matrix data that contain differentially assigned taxa and read counts for each category (greenhouse, wild, and magenta) were generated using the “dcast” function of the reshape2 package (66) and were rarefied using the “rrarefy” function in the vegan package (67).

We measured alpha diversity using the effective number of species (calculated as the exponential of the Shannon diversity) for the taxa assigned during metagenomic profiling. After checking the assumptions for parametric tests on the data, we used a simple analysis of variance (ANOVA) to test the differences in the effective number of species between the three host genotypes within the greenhouse-grown samples (Idaho, Nevada, and Utah) and between host phenotype among wild samples. We used the Kruskal-Wallis test to check for the difference between categories (greenhouse and wild samples) because the data failed the assumptions for a parametric test.

We calculated Bray-Curtis microbial community dissimilarities between samples using the “vegdist” function in the vegan package. Differences in community structure among host genotypes (Idaho, Nevada, and Utah) of the greenhouse-grown plants and phenotype-driven differences among wild plants were assessed using a permutational multivariate analysis of variance (PERMANOVA) on Bray-Curtis distances using the “adonis” function (also in the vegan package). Likewise, differences in microbial community composition between greenhouse-grown and field-grown plant categories were assessed using PERMANOVA on Bray-Curtis distances. For all PERMANOVA comparisons, we also tested for homogeneity of variance using the “betadisper” function.

#### Graphs

**Fig 2:**
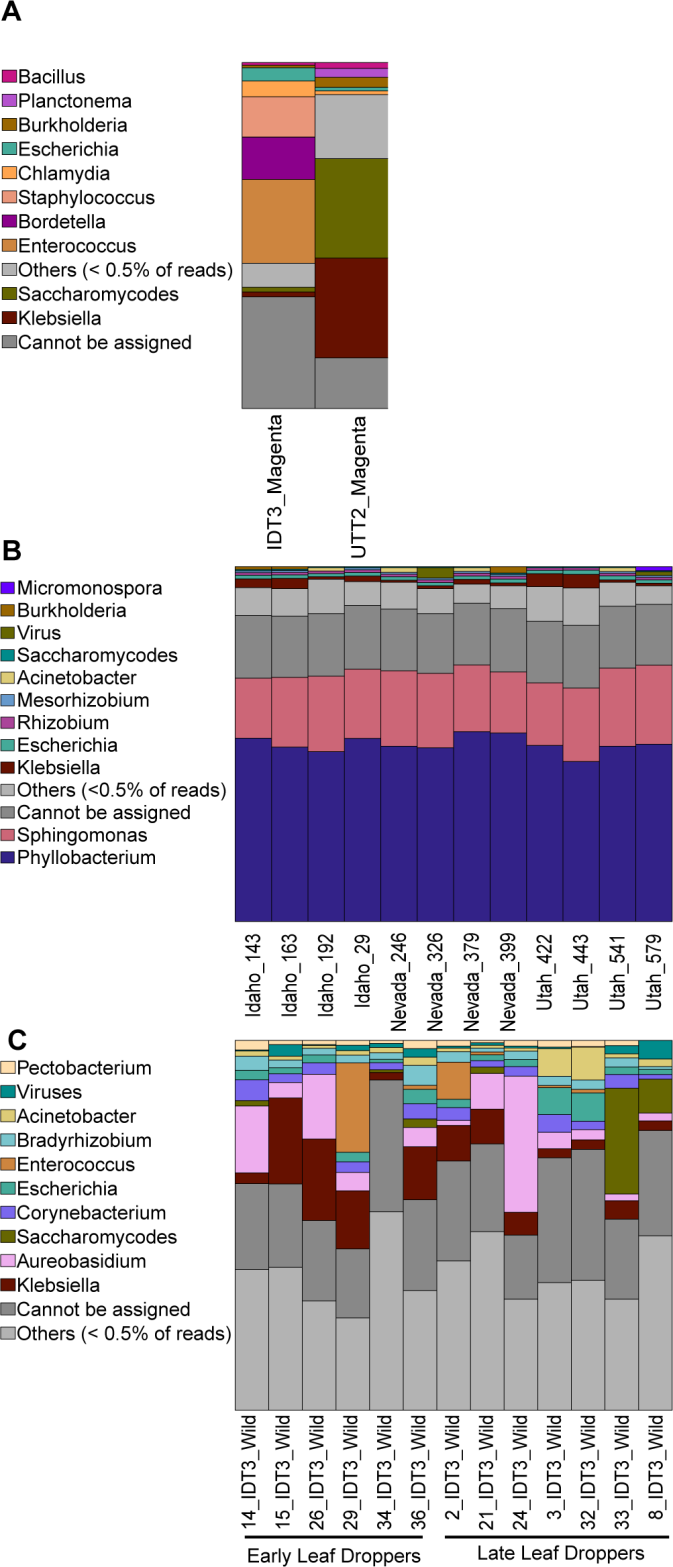
Taxonomic barplot showing the relative abundance of the top taxa at the genus level across (**A**) magenta box samples, (**B**) greenhouse samples, and (**C**) wild/field samples.

**Fig 3:**
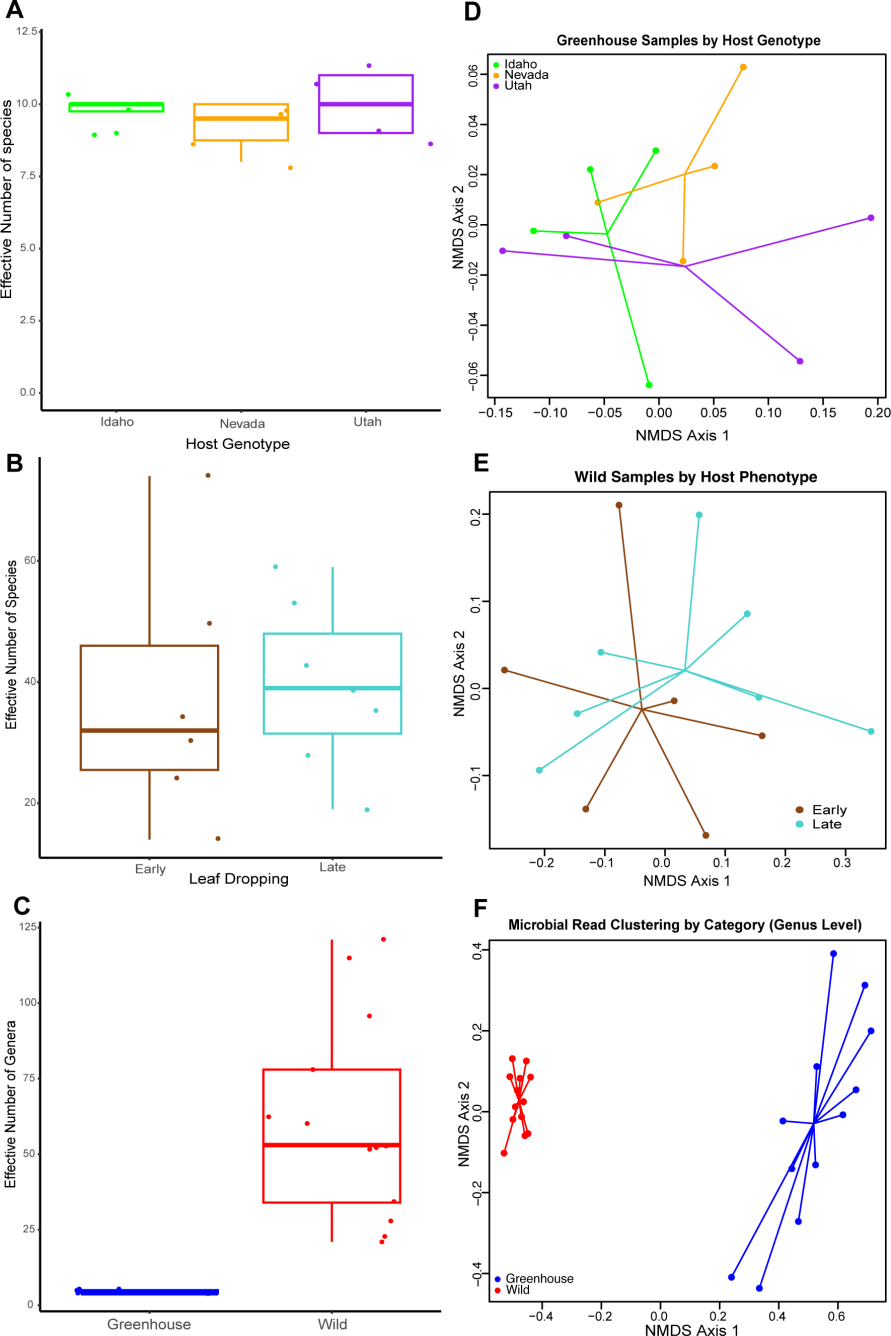
Wild samples had higher richness and different community composition than greenhouse samples. (**A**) Alpha diversity boxplot showing the effective number of species in greenhouse samples across genotypes from distinct origin populations. (**B**) Alpha diversity boxplot showing the effective number of species among wild samples with different leaf-dropping phenotypes. (**C**) Alpha diversity boxplot showing higher microbial diversity at the genus level in wild samples relative to greenhouse samples. (**D**) Non-metric multi-dimensional scaling (NMDS) plot showing no relationship between host genotype and microbial composition among the three populations of plants grown together in a greenhouse. (**E**) NMDS plot showing no relationship between host phenotype and microbial composition among wild samples. (**F**) NMDS plot showing strong clustering between microbial communities at the genus level in the categories of wild vs greenhouse plants.

**Fig 4:**
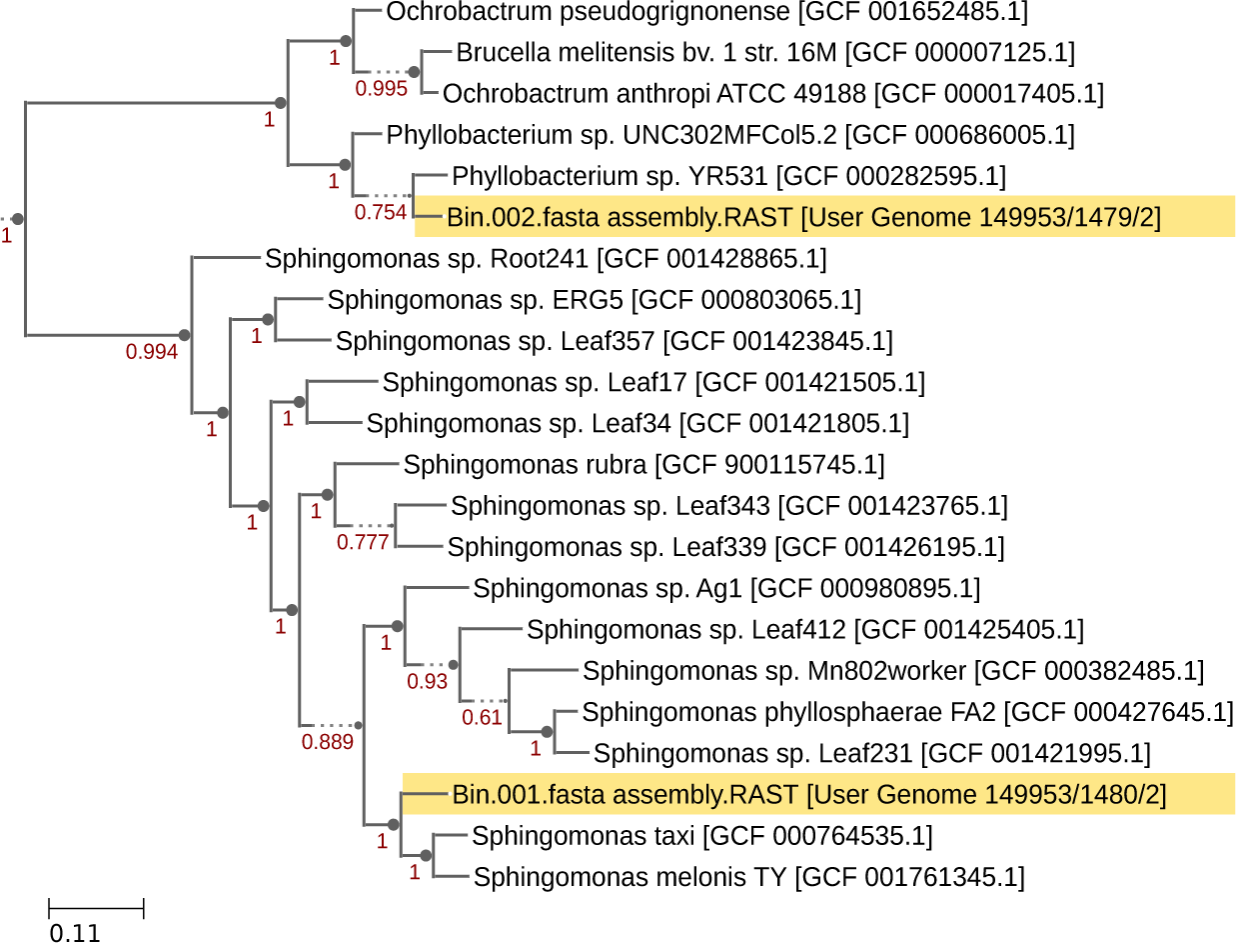
The placement of two high-quality MAGs in a phylogenomic tree supports their identities as a *Phyllobacterium* species and a *Sphingomonas* species.

**Fig 5:**
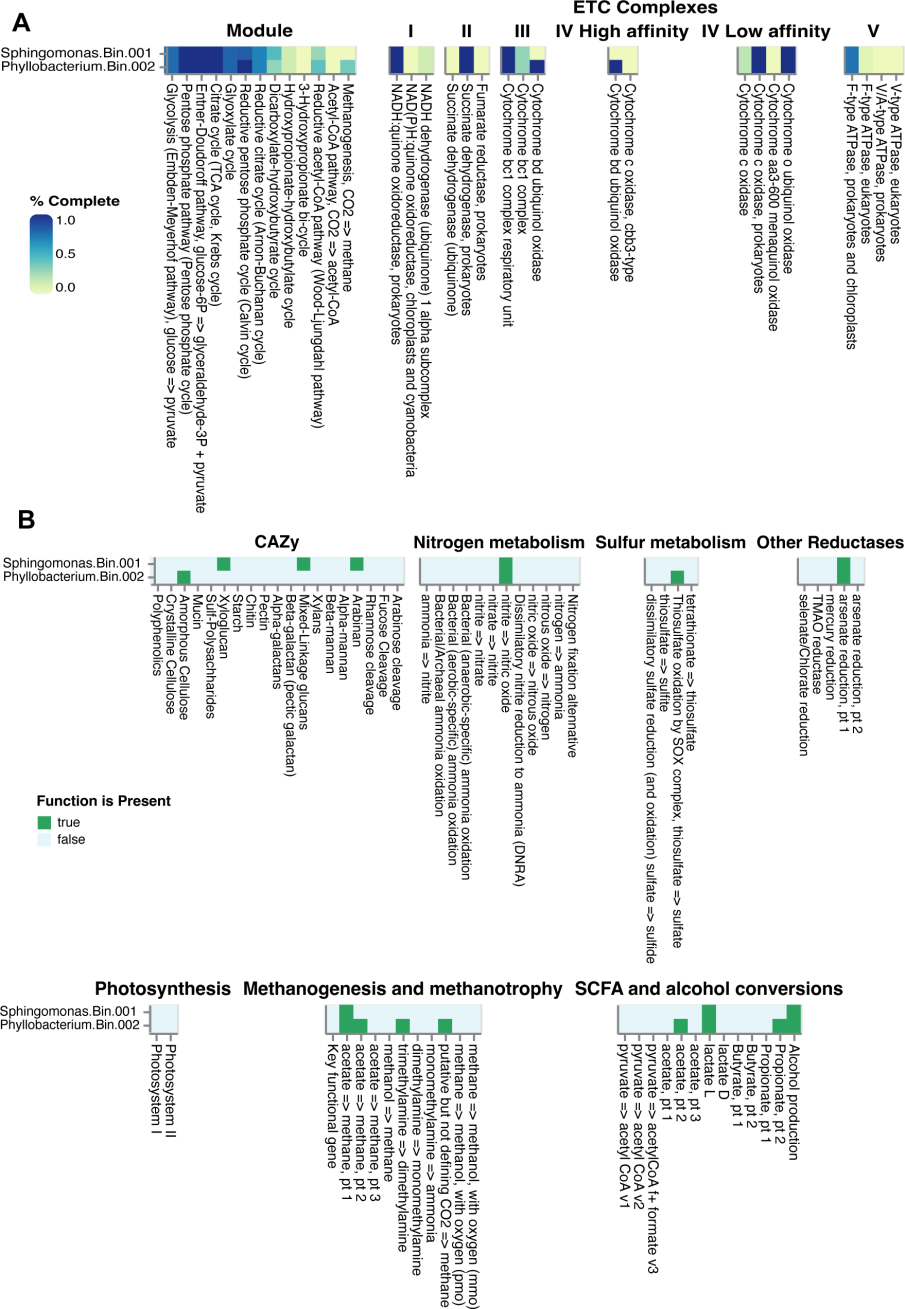
Functional annotation plots. (**A**) DRAM annotation showing the modules and electron transport chain complexes from the metagenomes. (**B**) Annotation of carbohydrate-active enzymes (CAZy) and metabolite utilization from the metagenomes.

Figure 1 was created using Microsoft PowerPoint. Figures 2 and 3 were plotted using R version 4.2.1 (65). Alpha and beta diversity figures were plotted using the “ggplot” function in the ggplot2 package (68). Taxonomy bar plots were plotted using the “barplot” function in base R. Figures 4 and 5 were generated using SpeciesTree (v.2.2.0) and the Annotate and Distill Assemblies with DRAM (v.0.1.2) pipeline on KBase (39), respectively.

## RESULTS

### Preprocessing (quality assessment and removal of host reads)

As expected, a large proportion of the initial reads were removed after mapping sequences to the *Artemisia tridentata* genome (Table 1), and we saw further clean-up across all samples after mapping against the closely related *A. annua* genome. We also removed human reads from magenta and wild samples, but no human reads were detected in the greenhouse samples (Table 1). The large differences in the sizes of “host”-cleaned reads, final read sizes, and the number of cleaned paired-end reads across the samples (especially with the two magenta samples relative to the wild and greenhouse samples) were due to differences in sequencing depth for these samples. Magenta samples were sequenced at ~160× sequencing depth, while greenhouse and wild samples were sequenced at ~20× and ~10–12×, respectively.

### Taxonomic assignment of short metagenome reads

Taxonomic profiling with different software packages (Kaiju, Kraken2, and DIAMOND) revealed that less than 20% of the reads across each sample could be classified, that is, assigned taxa, with the rest of the data not having a match across the different databases that were used. This might indicate that these unclassified reads were novel sequences; however, due to the complex nature of the sagebrush genome (with 78% being repetitive elements [20]), these are most likely to be repetitive host-related sequences that were not properly removed when mapping to the host genome and are not relevant for microbial analyses. In order to verify this, we re-mapped the “cleaned” reads from one sample back to the plant host reference genome again, and the result showed that many of the reads matched to regions of the plant genome at higher than 80% identity. This issue has also been seen in other studies (69–71).

The magenta box samples were dominated by human-associated microbes such as *Staphylococcus* sp., *Escherichia* sp., and *Enterococcus* sp., among others (Fig. 2A). These microbes were likely introduced while caring for the plants during cultivation or during handling prior to DNA extractions. Therefore, we excluded these two samples from further downstream analyses. We also excluded “Sample 35” of the wild samples from downstream analysis because it had a significant number of human-contaminated reads (Table 1) and was an outlier in terms of community composition.

The most abundant microbial genera across the greenhouse samples were *Phyllobacterium* and *Sphingomonas* (Fig. 2B). Species from these genera are known plant-growth promoters and are likely to be truly plant-associated. We also note that the greenhouse samples did not contain detectable human reads and so were likely to be less contaminated with human-associated microbes (Table 1). Other taxa in the greenhouse samples in low relative abundances included *Rhizobium* sp., *Mesorhizobium* sp., and *Acinetobacter* sp. (Fig. 2B).

As might be expected, wild, field-collected samples were much more variable than the greenhouse samples. The genera with the highest relative abundances across all samples were *Klebsiella* and *Aureobasidum*. Other studies of sagebrush leaf microbiomes have found *Aureobasidium* species, which are black, yeast-like fungi, at high abundances (25). Although the wild samples contained *Escherichia* sp., *Enterococcus* sp., and *Mycobacterium* sp., which are generally human-associated (Fig. 2C), the significant proportions of plant microbes such as *Acinetobacter* sp., *Aureobasidium* sp., and *Pseudomonas* sp. across the samples ensured that the majority of our taxa were true plant associates.

#### Alpha diversity

For greenhouse samples, which are composed of three host genotypes, the effective number of species per sample ranged from 8 to 11, with a median of 10 and a mean of 9.67. These samples were very similar and even, with no significant differences in the effective number of species among the host genotypes (ANOVA, *P*-value = 0.522) (Fig. 3A). Tukey’s HSD *post hoc* tests showed no significant pairwise differences between any of the genotypes (all adjusted *P*-values >0.50). Confidence intervals for mean differences all included zero. For wild samples, the effective number of species per sample ranged from 16 to 84, with a median of 42.5 and a mean of 45.43. There was no significant difference in the effective number of species among wild samples with different phenotypes (ANOVA, *P*-value = 0.861110) (Fig. 3B). Tukey’s HSD test also showed that the mean difference between the two phenotypes (9.86) was not significant, with a 95% confidence interval of (lwr = −19.69, upr = 39.40). However, there was higher microbial diversity (Kruskal-Wallis test, *P*-value = 1.544e − 05) in the wild plants relative to the greenhouse plants (Fig. 3C). Because only two groups (greenhouse and wild plants) were compared, this result is equivalent to a Wilcoxon rank-sum test, which confirmed that greenhouse plants exhibited significantly lower values than wild/field plants (W = 0, *P* = 1.75 × 10⁻⁵). The Hodges-Lehmann estimate indicated a median difference of −49 (95% CI: −74 to −30), which shows a significant separation between the groups.

#### Beta diversity

NMDS using Bray-Curtis dissimilarity and an associated PERMANOVA showed no relationship between microbial composition and plant host genotypes among greenhouse samples (*R*^2^ = 0.16036, *P*-value = 0.555, stress = 0.0382), also a non-significant (*P*-value = 0.5123) test for homogeneity of multivariate dispersions (betadisper) indicated that within-group variability was comparable across host genotypes. This is not surprising because of the close proximity and low microbial diversity of these plants while growing in the greenhouse (Fig. 3D).

Likewise, tests for homogeneity of multivariate dispersion were not significant (betadisper, *P*-value = 0.9887) for wild/field samples, indicating similar dispersion of microbial communities across host phenotypes. Consistent with this, PERMANOVA revealed no significant relationship between microbial community composition and host phenotype among wild plants (*R*^2^ = 0.08535, *P*-value = 0.437, stress = 0.0614; Fig. 3E).

In contrast, when comparing greenhouse and wild samples, tests for homogeneity of dispersion were significant (betadisper, *P*-value = 1.889e − 12), indicating differences in multivariate dispersion between environments. Although this suggests caution must be taken in interpreting PERMANOVA results, we noted clear separation of clusters (Fig. 3F), and a PERMANOVA conducted using Bray-Curtis distances nevertheless revealed a strong and significant difference in genus-level community composition between greenhouse and wild samples (*R*^2^ = 0.72119, *P*-value = 0.001, stress = 0.0652; Fig. 3F), suggesting substantial environmental structuring of the microbial communities between these two categories.

### Read assembly and reconstruction of MAGs

According to the Compare Assembled Contig Distributions and its metrics, metaSPAdes performed best because it produced the highest number of larger contigs that were between 25 and 75 Kb (Fig. S1). Therefore, we chose to move forward with it for our metagenomic binning. Although no bins were recovered from the wild sample co-assembly, metaSPAdes also performed better in that case.

We recovered three MAGs with >70% completion from the co-assembled contigs from the greenhouse samples. These MAGs were filtered using CheckM with set parameters of >50% completion and <10% contamination; this effectively removed one of the bins and retained two high-quality MAGs with >98% completion and <1% contamination/redundancy (Table 2). We recovered no bins from the co-assembly of the wild, field-collected samples. To check if this was due to the large variability in microbial composition and high diversity across these samples, we also tried assembling each wild sample individually. However, we got the same result when we assembled and binned the samples individually. Therefore, it is likely that the wild samples did not assemble well into potential MAGs due to the lower sequencing depth together with the higher diversity of these communities.

**TABLE 2:** CheckM quality assessment of MAGs that were recovered from greenhouse plants^*a*^

Bin name	Marker lineage	Genomes size (bp)	# Markers	# Marker sets	Completeness (%)	Contamination (%)
Bin.001	o__Sphingomonadales	3,972,965	569	293	98.62	0.9
Bin.002	c__Alphaproteobacteria	5,311,195	349	230	100	0.87
*Bin.003*	*k__Archaea*	*21,065,390*	*149*	*107*	*70.02*	*23.3*

^*a*^ The italicized text shows the one low-quality bin that was filtered out with the set parameter of >50% completion and <10% contamination.

### Taxonomic classification of recovered MAGs

The two high-quality MAGs were classified using GTDB-Tk v.1.7.0 of the GTDB as *Sphingomonas* sp. and *Phyllobacterium* sp. Corroborating this taxonomic assignment, phylogenomic placement using SpeciesTree v.2.2.0 placed these bins with related species as shown in Fig. 4.

### Functional annotation of genes

Our analyses using DRAM and Carbohydrate-Active Enzymes Database (CAZy) showed that our two recovered MAGs contain basic functions that are necessary for survival (Fig. 5). The MAGs have close to 100% completion for life-essential modules/pathways such as Embden-Meyerhof, Entner-Doudoroff, Pentose Phosphate, TCA cycle. They are also very similar in their annotations for the ETC (Electron Transport Chain) complexes, with the exception of Cytochrome bd ubiquinol oxidase, which is only found in Bin.002 (*Phyllobacterium*). Cytochrome bd is a ubiquinol:oxygen oxidoreductase of many prokaryotic respiratory chains, and it catalyzes the reduction of oxygen (O_2_) to water (H_2_O) by using quinols as a reducing substrate (72). It enables bacterial O_2_ consumption to promote specific metabolic pathways and/or to prevent O_2_ toxicity and other stress conditions, including high temperature and exposure to toxic compounds (73–75). KEGG annotation recovered multiple genes in our two MAGs associated with degradation pathways of chemicals found in sagebrush leaves (Table 3). We note that per-gene coverage for these degradation pathways was not calculated, but would increase confidence that these pathways were well-represented and functional in the genomes.

**TABLE 3:** KEGG annotation table showing the number of genes in categories related to the degradation of chemicals found in sagebrush leaves

KEGG pathway	Number of pathway genes found in MAGs		Subcategory related to degradation of sagebrush chemicals
	Bin.001 *Sphingomonas*	Bin.002 *Phyllobacterium*	
Geraniol degradation	16	16	K19653 geoA; geraniol dehydrogenase (NAD+) (EC:1.1.1.347)
Limonene and pinene degradation	12	12	K21569 camC, CYP101A1; camphor 5-monooxygenase (EC:1.14.15.1)
Nitrotoluene degradation	18	20	K21683 dntB; 4-methyl-5-nitrocatechol 5-monooxygenase (EC:1.14.13.210)

## DISCUSSION

Our culture-independent approach, using sagebrush high-throughput whole-genome sequencing to explore the leaf microbiome, was able to characterize associated taxa. Previous microbial metagenomics studies have highlighted how low coverage of microbial genomes and high microbial diversity can hinder the recovery of MAGs (76, 77). For example, one study recovered 20 MAGs from soil with 4.3 Gb of sequencing data (and soil is known to be one of the environments with the highest bacterial richness), while another identified 12 MAGs from olive metagenomes from 3.47 Gb of sequencing data, with the majority obtained from samples exhibiting higher average coverage and lower community complexity (78, 79). The sequencing depths were unequal across our sample types because the data used in this study originated from previous sagebrush studies conducted by researchers at Boise State University, which involved different initial sequencing efforts. According to Orellana et al. (80), differences in sequencing depth directly impact the analysis of microbial diversity and the successful recovery of MAGs because lower sequencing depth, especially when coupled with high microbial diversity, can impede the reconstruction of high-quality MAGs. In line with this, our results were best from the greenhouse-grown plant samples, which had deep enough host genome sequencing (~20×) and simple enough richness (~10 effective number of species per sample) to generate high-quality MAGs of the two most abundant bacterial taxa. The wild-collected samples, with ~10–12× host genome sequencing and much higher species richness, were too complex to build high-quality MAGs. This coverage and diversity information will likely be useful for other studies aiming to use plant host genome sequencing to better characterize the associated microbiome.

To the best of our knowledge, this is the first study to use this approach of mining plant genomes to generate microbial MAGs. Roman-Reyna et al. (17) used whole-genome-shotgun sequences from rice that encompassed both rice and the collective DNA of resident microbial communities to leverage the “unmapped” non-plant reads as microbiome data set. However, they did not generate MAGs. Numerous previous studies have used computational filtering of human genome-mapped reads, as it is a common preprocessing step in human microbiome research (81) and other studies on plant microbiomes have also cleaned metagenomes with the plant host genome. For example, Bulgarelli et al. (82) mapped metagenome samples to barley annotated genomic sequences using bwa-mem. In a more recent study, Regalado et al. (15) removed *Arabidopsis thaliana* reads from their metagenomic data by aligning against the *A. thaliana* reference genome and used only read pairs that were not mapped with the plant reference genome for classification.

One issue we faced is that only a small percentage of our post-host-removal reads were able to be classified. All of the classification programs we used showed that less than 20% of the reads across each sample could be classified. Further analysis suggested that many of these unclassified reads were host-associated repetitive sequences that were not properly removed when mapping against the host genome. However, having high numbers of unclassified reads is common in microbiome research, where multiple studies using shotgun data have found that more than 50% of reads cannot be annotated against known databases (15, 50, 77, 83). Regalado et al. (15) checked the efficiency of host-read removal by mapping the unclassified reads with *A. thaliana* reference genome, but less than 1% mapped with the reference genome, and they reported that only a small percentage of unclassified reads was likely to come from the plant. Thus, their unmapped reads were mostly from portions of microbial genomes not present in the NCBI nr database used with DIAMOND. This is likely partially the case for our study; however, the big sagebrush genome is one of the most highly repetitive plant genomes to be sequenced and assembled, with repeat elements comprising 77.99% of the genome (20) and it is very large when compared with *Arabidopsis thaliana* (~0.13 Gb for *A. thaliana* relative to ~4.2 Gb for *A. tridentata*). Therefore, in our study, many of these unmapped reads are likely to be host-derived and just not yet properly assembled into the highly complex host genome.

The most abundant taxa in all our greenhouse samples were *Phyllobacterium* and *Sphingomonas* species (Fig. 2B). These two taxa showed up reliably, and with very little variation across individual plants. Both genera contain known plant-growth-promoting species. *Phyllobacterium* is known plant-growth-promoting bacteria on several plants like *Picea* spp.*, Brassica napus*, and *Arabidopsis thaliana* (84, 85). Some *Phyllobacterium* species have been observed to enhance plant tolerance to abiotic stresses such as drought. Bresson et al. (86) reported that inoculation of *Phyllobacterium brassicacearum* strain STM196 improved the drought tolerance in *Arabidopsis thaliana* under a long-term water deficit. *Sphingomonas* are also known to produce indole acetic acid, gibberellins, and essential amino acids (87, 88). *Sphingomonas* species are known for their ability to degrade complex compounds such as aromatic hydrocarbons and other xenobiotics (89). Although their ability to degrade cellulose and hemicellulose could be beneficial for carbon decomposition and recycling, they can become opportunistic pathogens in certain conditions (90). To understand more about the capabilities of the strains we recovered, we annotated the functional genes of our MAGs. Analysis of carbohydrate-active enzymes highlighted differences in hydrolytic capabilities of the two bacteria, with the *Sphingomonas* species having the genes to degrade xyloglucan, mixed-linkage glucans, and arabinan while the *Phyllobacterium* species has genes for degrading amorphous cellulose (Fig. 5B). Interestingly, from our KEGG annotation results, we found that specific genes associated with the degradation of chemicals found in sagebrush leaves were abundant in our two MAGs (Table 3). Geraniol, limonene, and pinene are naturally occurring terpenes in sagebrush (63, 64). They contribute to the characteristic scent of the plant and potentially serve as protective agents against herbivores and pathogens. Therefore, it makes sense that these MAGs might contain genes capable of metabolizing these toxic compounds. Nitrotoluene has not been reported to be present in sagebrush; however, detoxification enzymes are often capable of metabolizing other substrates with similar chemical structures; therefore, it is possible that the products of these genes could degrade similar compounds that are found in sagebrush. Thus, a previous study by Kohl et al. (91) that investigated the genomic potential of microbes in the gut of sage-grouse fed with 100% sagebrush also looked into the presence of nitrotoluene-degrading genes. These chemicals are key parts of the complex sagebrush leaf chemistry and suggest that the *Phyllobacterium* and *Sphingomonas* species we recovered have the potential to metabolize secondary metabolites which are generally considered antimicrobial.

Relative to the greenhouse samples, the wild samples were more variable with high alpha diversity compared to the greenhouse plants (Fig. 3C). The reduced diversity of the greenhouse samples was likely because the plants were grown in the same controlled environment and were exposed to fewer microbial species and less environmental variability than they would be in their natural environment. Greenhouse-grown plants often have reduced leaf microbiomes (14), so it makes sense that our wild samples were more diverse. Our beta-diversity analyses also showed significant differences in community structure among greenhouse and wild samples (Fig. 3F). Several factors contribute to microbial community assembly in plants, including host genotype, where plant hosts exert some control over the abundance and composition of their microbiome as a result of the differing chemical and physical features of resources provided on their phyllosphere (92), a phenomenon known as genotype effects (12). The greenhouse-grown plants we used had different genotypes (34), but our results showed no significant relationship between microbial composition and plant host genotypes (Fig. 3D). This could be due to the reduced microbial diversity in the greenhouse, particularly for plants that had been grown in a sterile environment as seedlings before entering the greenhouse.

Our field-collected samples were likely all of similar genotype, but with different leaf-dropping phenotypes. While previous studies have shown that there is a significant relationship between microbial communities and plant phenotypes (93, 94), our test for phenotype-driven differences in the microbiome of these more natural samples showed no significant relationship (Fig. 3E).

Although taxonomic composition varied strongly across wild samples, the most abundant genera were *Klebsiella* and *Aureobasidum*. Recent research from the Bittleston lab group using both sequencing and culture-based methods to characterize fungal associates has found *Aureobasidium pullulans* to be the most abundant fungal species on sagebrush leaves (25). This cosmopolitan species often associates with plants (95–97) and can also be found in water or in harsh environments. It is a black yeast, and the high melanin content may provide some protection against UV light damage. *A. pullulans* produces siderophores that provide iron to plants and the antifungal pullulan that can inhibit pathogens (98, 99). Exopolysaccharides from *A. pullulans* can also increase soil water retention capacity and cation exchange capacity, which promotes nutrient availability and increases plant yield (100). The most abundant bacterium identified in our wild samples was *Klebsiella pneumoniae*, a common plant-associated bacterium capable of a broad range of functions such as nitrogen fixation, production of phytohormones like indole acetic acid (IAA), solubilization of inorganic phosphates, and compound degradation (101). Kumar et al. (102) reported that *K. pneumoniae* strain M6, which was isolated from mango rhizosphere, is a potent plant growth promoter, biocontrol, and paclobutrazol (a chemical that inhibits gibberellin biosynthesis in plants) degrading agent. Thus, both the *Klebsiella* and *Aureobasidium* taxa we identified here have the potential to benefit the sagebrush host plant.

### Conclusion

We used a culture-independent method to study the plant microbiome directly from host-targeted Illumina shotgun sequence data. As might be expected, wild samples were much more diverse than samples grown in a greenhouse and/or sterile magenta boxes. The top taxa from our greenhouse samples were *Phyllobacterium* sp. and *Sphingomonas* sp., while *Klebsiella* sp. and *Aureobasidium* sp. were the top taxa in wild samples. All these genera contain species known to support plant health. We recovered two high-quality MAGs from the co-assembly of the greenhouse samples, and functional annotation showed that these MAGs contain genes with the potential to degrade complex secondary compounds found in sagebrush leaves, further supporting the likelihood that these are long-term associations of this foundational plant species.

## Data Availability

All host-cleaned microbial sequences that were pulled out of the Illumina sequences have been deposited in the NCBI BioProject database under accession number PRJNA1256099. The complete analysis pipeline, data processing workflows, and reproducibility documentation, isare publicly available on Zenodo at: (https://doi.org/10.5281/zenodo.16601103).

## SUPPLEMENTAL MATERIAL

Supplemental material spectrum.02198-25-s0001.docx Details of other computational tools that we also used with our data; Fig. S1.

ASM does not own the copyrights to Supplemental Material that may be linked to, or accessed through, an article. The authors have granted ASM a non-exclusive, world-wide license to publish the Supplemental Material files. Please contact the corresponding author directly for reuse.
