# Supplementary material for: Exploring sagebrush leaf microbial metagenomes from deep, host-derived sequencing
Source: Microbiol Spectr. 2026 Mar 31;14(5):e02198-25. doi: 10.1128/spectrum.02198-25 (PMC13141870; doi:10.1128/spectrum.02198-25)
Supplement: Supplemental material — Details of other computational tools that we also used with our data; Fig. S1. [file spectrum.02198-25-s0001.docx]

# SUPPLEMENTARY INFORMATION

## Other Computational Tools

This section contains details of other computational tools that we also used with our data and the reason they were not included in our methods.

Taxonomic Assignment of short Illumina reads

We also explored the option of using SortMeRNA (Kopylova et al., 2012) to pull out ribosomal DNA (rDNA) from our metagenomic datasets and use QIIME2 (Bolyen et al., 2019) to classify our samples using rDNA data. We found that the result was not consistent with the results from Kaiju, Kraken2, and DIAMOND, for this reason, we did not include it as part of our workflow. MetaPhylAn (Beghini et al., 2023), a recently released tool for taxonomic profiling of metagenomic samples, was also tested on our dataset but was only able to identify *Sphingomonas* sp. from the reads across all the greenhouse samples. Albeit partial, the result from this tool was consistent with Kaiju, Kraken2, and DIAMOND. We think that the genetic markers for its database might need to be updated in order to generate a more complete profile. The partial profiling with the greenhouse samples prevented us from using it with our sequencing data from wild and magenta samples and therefore MetaPhylAn was not included in our workflow.

Metagenome Assembly and Binning

We also tried Anvi’o co-assembly (Eren et al., 2015) which generated 5 bins but taxonomy showed that the majority of the binned contigs are from the host plants and not microbial reads. We also independently assembled each sample using the recently released MetaBinner (Wang et al., 2023) which produced an average of 3 bins/sample but with very low quality when assessed with CheckM. Although we used one of Anvi’o’s modules for KEGG annotations of bins from Kbase, Anvi’o co-assembly and MetaBinner were not included in our pipeline.

Gene Prediction/Functional Profiling of MAGs

We also used the ‘anvi-run-kegg-kofams’ module of the Anvi’o analysis platform to produce KEGG annotations of the MAGS which were comparable with the output of KofamScan. Also, due to the interesting nature of sagebrush chemistry, we checked the MAGs for the production of secondary metabolites using the antiSMASH online predictive platform (Medema et al., 2011).

## Supplemental Figure


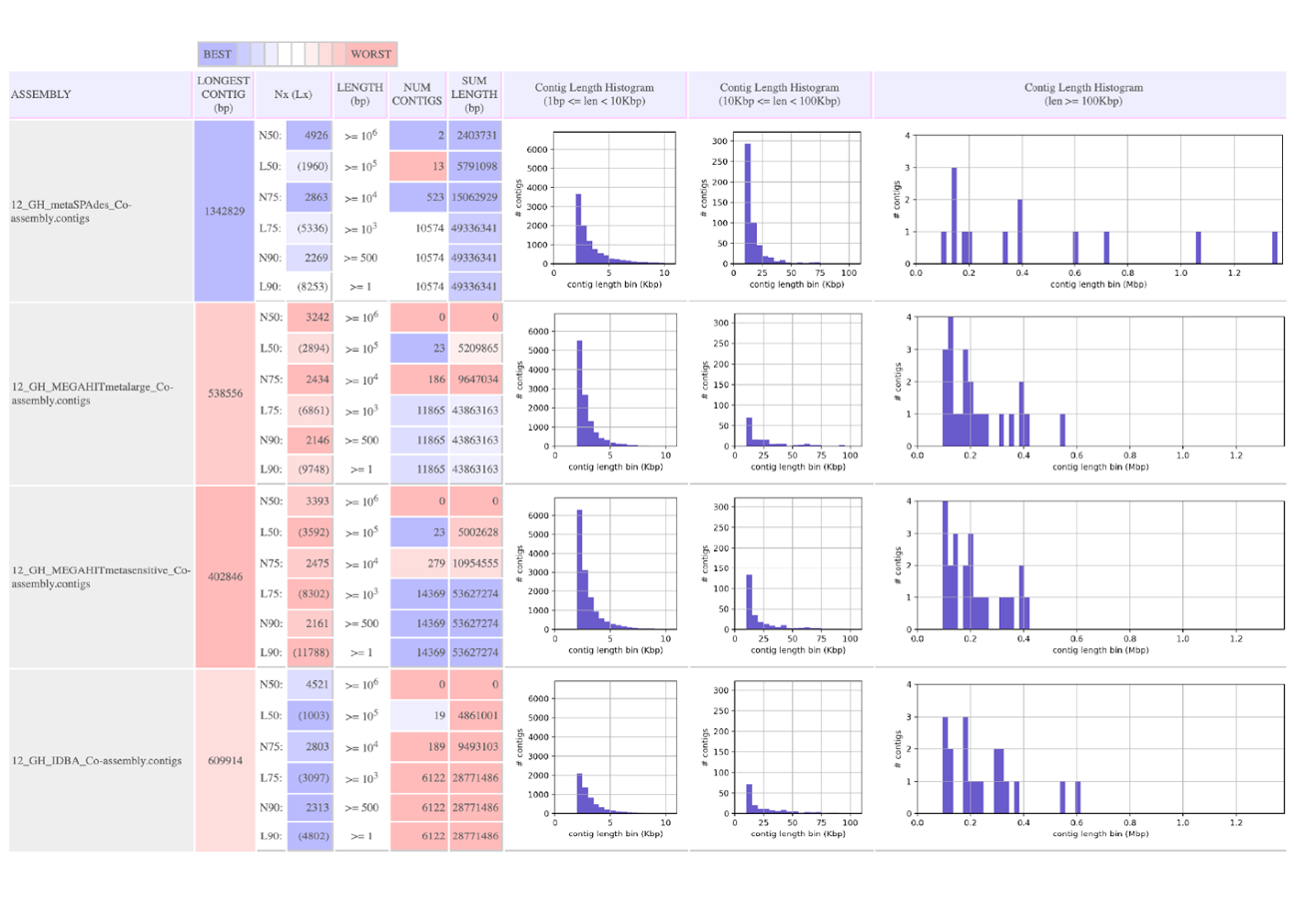


**Supplementary Figure 1: Contig Statistics. Plots showing the number of contigs, longest contigs, N50, L50, N75 etc. obtained from the binning tools that were used in this study.**
